# Bis[bis­(penta­methyl­cyclo­penta­dien­yl)cobalt(III)] tetra­chlorido­cobaltate(II) di­chloro­methane disolvate

**DOI:** 10.1107/S160053681302254X

**Published:** 2013-08-17

**Authors:** Joseph S. Merola, Mai Ngo, George W. Karpin

**Affiliations:** a Department of Chemistry, Virginia Tech, Blacksburg, VA 24061, USA

## Abstract

The title compound, [Co(C_10_H_15_)_2_]_2_[CoCl_4_]·2CH_2_Cl_2_, was isolated as a dichloromethane solvate and was formed in the reaction between lithium penta­methyl­cyclo­penta­dienide and anyhydrous cobalt(II) chloride in tetra­hydro­furan. There are two deca­methyl­cobaltocenium cations, one tetrachloridocobaltate(II) anion and two di­chloro­methane solvent mol­ecules in the formula unit. There is a slight disorder of the di­chloro­methane solvent which was treated with a two-site model [occupancy rates = 0.765 (4) and 0.235 (4)]. The di­chloro­methane mol­ecules display significant C—H⋯Cl inter­actions with the tetrachloridocobaltate(II) dianion. The cobalt atom of the deca­methyl­cobaltocenium cation sits on a twofold rotation axis, with only one penta­methyl­cyclo­penta­diene ligand being unique and the second generated by symmetry. The cobalt atom of the [CoCl_4_]^−2^ ion sits on a special site with -4 symmetry, with one unique chloride ligand and the others generated by the fourfold inversion axis.

## Related literature

For a related structure with a (THF)_2_LiCl_2_CoCl_2_ monoanion and the deca­methyl­cobaltocenium cation, see: Dehnen & Zimmermann (2000 ▶) (CCDC 135478). The structure of a related dimer synthesized by Koelle *et al.* (1986 ▶) was determined by Olson & Dahl (1986 ▶) (CCDC 566220). For a discussion of the role of chloro­form and di­chloro­methane solvent mol­ecules in crystal packing, see: Allen *et al.* (2013 ▶).
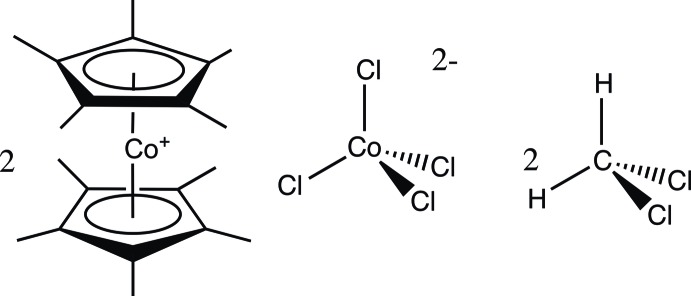


## Experimental

### 

#### Crystal data

[Co(C_10_H_15_)_2_]_2_[CoCl_4_]·2CH_2_Cl_2_
*M* *_r_* = 1033.35
Tetragonal, 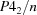

*a* = 12.20980 (12) Å
*c* = 16.2811 (3) Å
*V* = 2427.17 (7) Å^3^
*Z* = 2
Mo *K*α radiation
μ = 1.48 mm^−1^
*T* = 101 K
0.27 × 0.24 × 0.18 mm

#### Data collection

Agilent Xcalibur Gemini Ultra diffractometer with Eos detector
Absorption correction: gaussian (*CrysAlis PRO*; Agilent, 2013 ▶) *T*_min_ = 0.755, *T*_max_ = 0.821
26858 measured reflections
4161 independent reflections
3394 reflections with *I* > 2σ(*I*)
*R*_int_ = 0.037

#### Refinement

*R*[*F* ^2^ > 2σ(*F* ^2^)] = 0.037
*wR*(*F* ^2^) = 0.091
*S* = 1.04
4161 reflections
130 parameters
H-atom parameters constrained
Δρ_max_ = 1.21 e Å^−3^
Δρ_min_ = −1.07 e Å^−3^

### 

Data collection: *CrysAlis PRO* (Agilent, 2013 ▶); cell refinement: *CrysAlis PRO*; data reduction: *CrysAlis PRO*; program(s) used to solve structure: *SHELXS97* (Sheldrick, 2008 ▶); program(s) used to refine structure: *SHELXL2013* (Sheldrick, 2008 ▶); molecular graphics: *OLEX2* (Dolomanov *et al.*, 2009 ▶); software used to prepare material for publication: *OLEX2*.

## Supplementary Material

Crystal structure: contains datablock(s) I. DOI: 10.1107/S160053681302254X/zl2564sup1.cif

Structure factors: contains datablock(s) I. DOI: 10.1107/S160053681302254X/zl2564Isup2.hkl

Additional supplementary materials:  crystallographic information; 3D view; checkCIF report

## Figures and Tables

**Table 1:** Hydrogen-bond geometry (Å, °)

*D*—H⋯*A*	*D*—H	H⋯*A*	*D*⋯*A*	*D*—H⋯*A*
C11—H⋯Cl1^i^	0.97	2.71	3.548 (3)	145
C11—H*A*⋯Cl1^ii^	0.97	2.71	3.548 (3)	145

Symmetry codes: (i) 
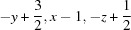
; (ii) 
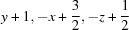
.

**Fig. 1.**
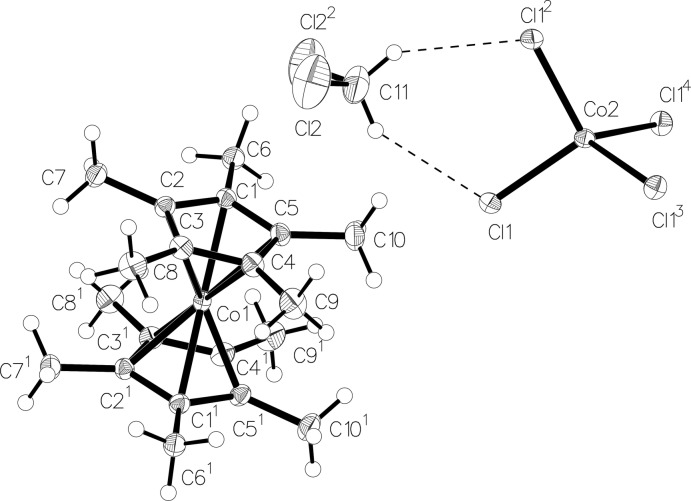
Plot of the title compound displaying the complete molecular fragments with thermal ellipsoids shown at 50% probability level. Only the major component (76.3 (4)%) of the disordered dichloromethane solvate is shown. Symmetry codes: (1) 3/2 - x, 1/2 - y, z; (2) 3/2 - x, 3/2 - y, z; (3) y, 3/2 - x, 3/2 - z; (4) 3/2 - y, x, 3/2 - z

**Fig. 2.**
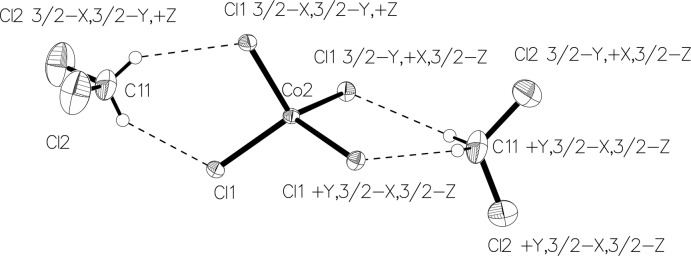
Thermal ellipsoid plot of the CoCl4-2 ion showing C—H···Cl interactions with the dichloromethane solvate. Atoms are named with the symmetry operation that generated them. Only the major component (76.3 (4)%) of the disordered dichloromethane solvate is shown.

## supplementary crystallographic information

### 1. Comment

Koelle *et al.* (1986) reported on the preparation of a pentamethylcyclopentadienylcobalt chloro-bridged dimeric compound that represents an excellent precursor to other mononuclear complexes *via* bridge-splitting reactions. There is no doubt that Koelle *et al.* made the described bridged complex since Olson & Dahl (1986) published a crystal structure of the dimer. However, in our laboratories, regardless of the stoichiometry used in attempts to make the chloro bridged dimer described by Koelle *et al.* (1986), the title compound was the only material isolated. The composition of the title compound consists of two decamethylcobaltocenium cations, one tetrachlorocobalt(II) dianion, and two molecules of dichloromethane. The dichloromethane molecules were slightly disordered and the disorder was treated successfully by a two site model with occupancies of 76.5 (4) and 23.5 (4)%. Dehnen & Zimmerman (2000) noted a similar product in attempts to make selenium-bridged compounds and noted the decamethylcoblatocenium ion was formed depending on the temperature of the reaction. In their case, the CoCl_4_^-2^ ion was linked *via* chloride bridges to a bis-THF Li^+^ cation.

In the structure reported here, the CoCl_4_^-2^ ion shows significant interaction with the dichloromethane of solvation. Recently, Allen *et al.* (2013) examined the Cambridge Crystallographic Data Base and analyzed crystallographic evidence of C—H···Cl hydrogen bonding for both CH_2_Cl_2_ and CHCl_3_. In that paper, for the specific case of CH_2_Cl_2_ interacting with Cl-, they note C—H···Cl interactions with H···Cl distances ranging from 2.33 to 2.95 Å and C—H···Cl angles ranging from 120° to 170° for a set of 63 structures. In the title structure, the H···Cl distance is 2.72 Å with a C—H···Cl angle of 145.0°. These parameters would place the C—H···Cl interaction for the title structure very nearly at the median of the structures analyzed by Allen *et al.* (2013).

In the structure reported here, the CoCl_4_^2-^ ion is also distorted from perfect tetrahedral geometry with the Cl—Co—Cl angles involved in the hydrogen bonding compressed to 100.04 (2)° and the remaining Cl—Co—Cl angles are 114.385 (12)°. While it is important not to make too much of a qualitative observation, the strength of the C—H···Cl interaction may also be responsible for the relative stability of these crystals in open air. Our experience is that, in the abence of a significant attractive interaction, dichlromethane molecules quite easily evaporate from crystals with loss of crystallinity at room temperature.

### 2. Experimental

The procedure described by Koelle *et al.*(1986) was followed using lithium pentamethylcyclopentadienide (LiCp*) and anhydrous cobalt(II) chloride in tetrahydrofuran. Instead of obtaining the hexane soluble brown dimer as described, the reaction produced a green solid. Dissolution of the solid in dichloromethane followed by slow diffusion of diethyl ether produced well formed green prisms of the title compound that are very air stable and retain the dichloromethane of solvation even after several weeks exposure to the open atmosphere at room temperature.

### Crystal data

**Table:**

[Co(C_10_H_15_)_2_]_2_[CoCl_4_]·2CH_2_Cl_2_	*D*_x_ = 1.408 Mg m^−^^3^
*M**_r_* = 1029.32	Mo *K*α radiation, λ = 0.7107 Å
Tetragonal, *P*4_2_/*n*	Cell parameters from 8251 reflections
*a* = 12.20980 (12) Å	θ = 4.2–32.2°
*c* = 16.2811 (3) Å	µ = 1.48 mm^−^^1^
*V* = 2427.17 (7) Å^3^	*T* = 101 K
*Z* = 2	Prism, clear green
*F*(000) = 1066	0.27 × 0.24 × 0.18 mm

### Data collection

**Table:**

Agilent Xcalibur (Eos, Gemini ultra) diffractometer	4161 independent reflections
Radiation source: Enhance (Mo) X-ray Source	3394 reflections with *I* > 2σ(*I*)
Graphite monochromator	*R*_int_ = 0.037
Detector resolution: 16.0122 pixels mm^-1^	θ_max_ = 32.5°, θ_min_ = 3.4°
ω scans	*h* = −18→17
Absorption correction: gaussian (*CrysAlis PRO*; Agilent, 2013)	*k* = −18→12
*T*_min_ = 0.755, *T*_max_ = 0.821	*l* = −24→21
26858 measured reflections	

### Refinement

**Table:**

Refinement on *F*^2^	Primary atom site location: heavy-atom method
Least-squares matrix: full	Secondary atom site location: difference Fourier map
*R*[*F*^2^ > 2σ(*F*^2^)] = 0.037	Hydrogen site location: constr
*wR*(*F*^2^) = 0.091	H-atom parameters constrained
*S* = 1.04	*w* = 1/[σ^2^(*F*_o_^2^) + (0.0319*P*)^2^ + 2.9104*P*] where *P* = (*F*_o_^2^ + 2*F*_c_^2^)/3
4161 reflections	(Δ/σ)_max_ = 0.001
130 parameters	Δρ_max_ = 1.21 e Å^−^^3^
0 restraints	Δρ_min_ = −1.07 e Å^−^^3^

### Special details

**Table:**

Geometry. All e.s.d.'s (except the e.s.d. in the dihedral angle between two l.s. planes) are estimated using the full covariance matrix. The cell e.s.d.'s are taken into account individually in the estimation of e.s.d.'s in distances, angles and torsion angles; correlations between e.s.d.'s in cell parameters are only used when they are defined by crystal symmetry. An approximate (isotropic) treatment of cell e.s.d.'s is used for estimating e.s.d.'s involving l.s. planes.
Refinement. Refinement of *F*^2^ against ALL reflections. The weighted *R*-factor *wR* and goodness of fit *S* are based on *F*^2^, conventional *R*-factors *R* are based on *F*, with *F* set to zero for negative *F*^2^. The threshold expression of *F*^2^ > σ(*F*^2^) is used only for calculating *R*-factors(gt) *etc*. and is not relevant to the choice of reflections for refinement. *R*-factors based on *F*^2^ are statistically about twice as large as those based on *F*, and *R*- factors based on ALL data will be even larger.

### Fractional atomic coordinates and isotropic or equivalent isotropic displacement parameters (Å^2^)

**Table:**

	*x*	*y*	*z*	*U*_iso_*/*U*_eq_	Occ. (<1)
Co1	0.7500	0.2500	0.361342 (19)	0.01381 (8)	
C1	0.86792 (13)	0.37038 (14)	0.36190 (10)	0.0171 (3)	
C2	0.79973 (13)	0.38033 (14)	0.29066 (10)	0.0169 (3)	
C3	0.68885 (14)	0.39523 (14)	0.31700 (11)	0.0193 (3)	
C4	0.68838 (14)	0.39480 (15)	0.40471 (11)	0.0206 (3)	
C5	0.79870 (14)	0.37984 (14)	0.43257 (10)	0.0188 (3)	
C6	0.98932 (14)	0.35672 (16)	0.36341 (11)	0.0221 (3)	
H6A	1.0095	0.3102	0.4085	0.033*	
H6B	1.0235	0.4270	0.3699	0.033*	
H6C	1.0131	0.3242	0.3128	0.033*	
C7	0.83672 (15)	0.38171 (16)	0.20305 (11)	0.0231 (4)	
H7A	0.9052	0.3436	0.1984	0.035*	
H7B	0.8459	0.4561	0.1853	0.035*	
H7C	0.7828	0.3464	0.1693	0.035*	
C8	0.59249 (15)	0.41374 (17)	0.26198 (13)	0.0268 (4)	
H8A	0.5987	0.3678	0.2144	0.040*	
H8B	0.5906	0.4891	0.2452	0.040*	
H8C	0.5263	0.3962	0.2910	0.040*	
C9	0.59133 (16)	0.41372 (19)	0.45881 (14)	0.0318 (4)	
H9A	0.5256	0.3966	0.4292	0.048*	
H9B	0.5896	0.4891	0.4756	0.048*	
H9C	0.5965	0.3676	0.5064	0.048*	
C10	0.83534 (17)	0.38163 (18)	0.52022 (12)	0.0281 (4)	
H10A	0.7814	0.3462	0.5539	0.042*	
H10B	0.8443	0.4561	0.5379	0.042*	
H10C	0.9039	0.3437	0.5251	0.042*	
Co2	1.2500	0.2500	0.2500	0.01550 (10)	
Cl1	1.11315 (3)	0.29420 (4)	0.15956 (3)	0.02050 (9)	
C11	1.2500	0.2500	0.5298 (2)	0.0505 (9)	
H	1.2150	0.1964	0.4946	0.061*	0.3825 (19)
HA	1.2850	0.3036	0.4946	0.061*	0.3825 (19)
HB	1.2469	0.1859	0.4946	0.061*	0.1175 (19)
HC	1.2531	0.3141	0.4946	0.061*	0.1175 (19)
Cl2	1.35262 (12)	0.1830 (2)	0.58797 (8)	0.0726 (5)	0.765 (4)
Cl2A	1.3635 (4)	0.2446 (6)	0.5851 (3)	0.0726 (5)	0.235 (4)

### Atomic displacement parameters (Å^2^)

**Table:**

	*U*^11^	*U*^22^	*U*^33^	*U*^12^	*U*^13^	*U*^23^
Co1	0.01106 (14)	0.01787 (15)	0.01251 (14)	0.00152 (11)	0.000	0.000
C1	0.0155 (7)	0.0193 (7)	0.0167 (7)	−0.0010 (6)	−0.0006 (6)	−0.0008 (6)
C2	0.0162 (7)	0.0183 (7)	0.0163 (7)	0.0004 (6)	−0.0001 (6)	0.0012 (6)
C3	0.0172 (7)	0.0186 (7)	0.0221 (8)	0.0032 (6)	−0.0017 (6)	−0.0003 (6)
C4	0.0186 (8)	0.0208 (8)	0.0224 (8)	0.0032 (6)	0.0015 (6)	−0.0042 (6)
C5	0.0177 (7)	0.0217 (8)	0.0171 (7)	−0.0002 (6)	0.0007 (6)	−0.0042 (6)
C6	0.0151 (7)	0.0317 (9)	0.0195 (8)	−0.0029 (7)	−0.0005 (6)	0.0007 (7)
C7	0.0219 (8)	0.0304 (9)	0.0169 (8)	−0.0021 (7)	0.0005 (6)	0.0031 (7)
C8	0.0191 (8)	0.0298 (9)	0.0316 (10)	0.0068 (7)	−0.0050 (7)	0.0037 (8)
C9	0.0223 (9)	0.0390 (11)	0.0342 (11)	0.0054 (8)	0.0083 (8)	−0.0124 (9)
C10	0.0272 (9)	0.0399 (11)	0.0171 (8)	−0.0027 (8)	−0.0007 (7)	−0.0060 (8)
Co2	0.01459 (13)	0.01459 (13)	0.0173 (2)	0.000	0.000	0.000
Cl1	0.01695 (18)	0.0238 (2)	0.02071 (18)	0.00320 (14)	−0.00162 (14)	−0.00033 (15)
C11	0.056 (2)	0.069 (3)	0.0266 (16)	0.000 (2)	0.000	0.000
Cl2	0.0681 (7)	0.0970 (14)	0.0527 (5)	0.0388 (9)	−0.0021 (5)	0.0055 (8)
Cl2A	0.0681 (7)	0.0970 (14)	0.0527 (5)	0.0388 (9)	−0.0021 (5)	0.0055 (8)

### Geometric parameters (Å, º)

**Table:**

Co1—C1^i^	2.0576 (17)	C7—H7B	0.9600
Co1—C1	2.0576 (17)	C7—H7C	0.9600
Co1—C2^i^	2.0556 (17)	C8—H8A	0.9600
Co1—C2	2.0556 (17)	C8—H8B	0.9600
Co1—C3	2.0550 (17)	C8—H8C	0.9600
Co1—C3^i^	2.0550 (17)	C9—H9A	0.9600
Co1—C4^i^	2.0470 (17)	C9—H9B	0.9600
Co1—C4	2.0470 (17)	C9—H9C	0.9600
Co1—C5^i^	2.0521 (17)	C10—H10A	0.9600
Co1—C5	2.0522 (17)	C10—H10B	0.9600
C1—C2	1.433 (2)	C10—H10C	0.9600
C1—C5	1.432 (2)	Co2—Cl1^ii^	2.2915 (4)
C1—C6	1.492 (2)	Co2—Cl1^iii^	2.2915 (4)
C2—C3	1.432 (2)	Co2—Cl1	2.2915 (4)
C2—C7	1.496 (2)	Co2—Cl1^iv^	2.2915 (4)
C3—C4	1.428 (3)	C11—H	0.9700
C3—C8	1.496 (2)	C11—HA	0.9700
C4—C5	1.433 (2)	C11—HB	0.9700
C4—C9	1.494 (3)	C11—HC	0.9700
C5—C10	1.496 (3)	C11—Cl2^iii^	1.771 (2)
C6—H6A	0.9600	C11—Cl2	1.771 (2)
C6—H6B	0.9600	C11—Cl2A^iii^	1.654 (5)
C6—H6C	0.9600	C11—Cl2A	1.654 (5)
C7—H7A	0.9600		
			
C1—Co1—C1^i^	179.49 (9)	C9—C4—Co1	128.93 (14)
C2—Co1—C1^i^	139.66 (7)	C1—C5—Co1	69.81 (9)
C2^i^—Co1—C1^i^	40.78 (6)	C1—C5—C4	108.10 (15)
C2—Co1—C1	40.78 (6)	C1—C5—C10	126.23 (16)
C2^i^—Co1—C1	139.66 (7)	C4—C5—Co1	69.34 (10)
C2—Co1—C2^i^	111.91 (9)	C4—C5—C10	125.54 (16)
C3—Co1—C1^i^	111.33 (7)	C10—C5—Co1	129.57 (14)
C3^i^—Co1—C1^i^	68.86 (7)	C1—C6—H6A	109.5
C3^i^—Co1—C1	111.33 (7)	C1—C6—H6B	109.5
C3—Co1—C1	68.86 (7)	C1—C6—H6C	109.5
C3^i^—Co1—C2	111.35 (7)	H6A—C6—H6B	109.5
C3—Co1—C2	40.77 (7)	H6A—C6—H6C	109.5
C3—Co1—C2^i^	111.35 (7)	H6B—C6—H6C	109.5
C3^i^—Co1—C2^i^	40.77 (7)	C2—C7—H7A	109.5
C3—Co1—C3^i^	138.87 (10)	C2—C7—H7B	109.5
C4^i^—Co1—C1	110.99 (7)	C2—C7—H7C	109.5
C4—Co1—C1	68.82 (7)	H7A—C7—H7B	109.5
C4—Co1—C1^i^	110.99 (7)	H7A—C7—H7C	109.5
C4^i^—Co1—C1^i^	68.82 (7)	H7B—C7—H7C	109.5
C4^i^—Co1—C2^i^	68.47 (7)	C3—C8—H8A	109.5
C4^i^—Co1—C2	138.86 (7)	C3—C8—H8B	109.5
C4—Co1—C2	68.47 (7)	C3—C8—H8C	109.5
C4—Co1—C2^i^	138.86 (7)	H8A—C8—H8B	109.5
C4^i^—Co1—C3	179.56 (8)	H8A—C8—H8C	109.5
C4—Co1—C3	40.75 (7)	H8B—C8—H8C	109.5
C4^i^—Co1—C3^i^	40.74 (7)	C4—C9—H9A	109.5
C4—Co1—C3^i^	179.56 (8)	C4—C9—H9B	109.5
C4^i^—Co1—C4	139.65 (11)	C4—C9—H9C	109.5
C4^i^—Co1—C5^i^	40.92 (7)	H9A—C9—H9B	109.5
C4^i^—Co1—C5	111.46 (7)	H9A—C9—H9C	109.5
C4—Co1—C5^i^	111.46 (7)	H9B—C9—H9C	109.5
C4—Co1—C5	40.92 (7)	C5—C10—H10A	109.5
C5—Co1—C1	40.79 (7)	C5—C10—H10B	109.5
C5^i^—Co1—C1	138.77 (7)	C5—C10—H10C	109.5
C5^i^—Co1—C1^i^	40.79 (7)	H10A—C10—H10B	109.5
C5—Co1—C1^i^	138.77 (7)	H10A—C10—H10C	109.5
C5—Co1—C2^i^	179.55 (7)	H10B—C10—H10C	109.5
C5^i^—Co1—C2^i^	68.45 (7)	Cl1^iii^—Co2—Cl1^ii^	114.385 (12)
C5^i^—Co1—C2	179.55 (7)	Cl1^iii^—Co2—Cl1	100.04 (2)
C5—Co1—C2	68.45 (7)	Cl1^ii^—Co2—Cl1	114.385 (11)
C5^i^—Co1—C3	139.45 (7)	Cl1^iii^—Co2—Cl1^iv^	114.386 (11)
C5—Co1—C3	68.73 (7)	Cl1^ii^—Co2—Cl1^iv^	100.04 (2)
C5—Co1—C3^i^	139.45 (7)	Cl1—Co2—Cl1^iv^	114.385 (11)
C5^i^—Co1—C3^i^	68.72 (7)	H—C11—HA	107.5
C5^i^—Co1—C5	111.19 (10)	H—C11—HC	102.2
C2—C1—Co1	69.54 (9)	HA—C11—HB	102.2
C2—C1—C6	126.87 (15)	HB—C11—HC	107.6
C5—C1—Co1	69.40 (10)	Cl2^iii^—C11—H	108.4
C5—C1—C2	107.49 (14)	Cl2—C11—H	108.4
C5—C1—C6	125.60 (15)	Cl2^iii^—C11—HA	108.4
C6—C1—Co1	127.98 (13)	Cl2—C11—HA	108.4
C1—C2—Co1	69.69 (9)	Cl2^iii^—C11—HB	131.4
C1—C2—C7	126.67 (15)	Cl2—C11—HB	88.3
C3—C2—Co1	69.59 (10)	Cl2^iii^—C11—HC	88.3
C3—C2—C1	108.52 (14)	Cl2—C11—HC	131.4
C3—C2—C7	124.72 (15)	Cl2—C11—Cl2^iii^	115.3 (2)
C7—C2—Co1	129.17 (13)	Cl2A^iii^—C11—H	88.8
C2—C3—Co1	69.64 (9)	Cl2A—C11—H	131.6
C2—C3—C8	125.70 (16)	Cl2A^iii^—C11—HA	131.6
C4—C3—Co1	69.33 (10)	Cl2A—C11—HA	88.8
C4—C3—C2	107.63 (15)	Cl2A—C11—HB	108.7
C4—C3—C8	126.60 (16)	Cl2A^iii^—C11—HB	108.7
C8—C3—Co1	128.79 (13)	Cl2A—C11—HC	108.7
C3—C4—Co1	69.93 (10)	Cl2A^iii^—C11—HC	108.7
C3—C4—C5	108.25 (15)	Cl2A—C11—Cl2^iii^	108.7 (2)
C3—C4—C9	126.30 (17)	Cl2A^iii^—C11—Cl2	108.7 (2)
C5—C4—Co1	69.73 (10)	Cl2A—C11—Cl2A^iii^	114.1 (4)
C5—C4—C9	125.35 (17)		

Symmetry codes: (i) −*x*+3/2, −*y*+1/2, *z*; (ii) *y*+1, −*x*+3/2, −*z*+1/2; (iii) −*x*+5/2, −*y*+1/2, *z*; (iv) −*y*+3/2, *x*−1, −*z*+1/2.

### Hydrogen-bond geometry (Å, º)

**Table:**

*D*—H···*A*	*D*—H	H···*A*	*D*···*A*	*D*—H···*A*
C11—H···Cl1^iv^	0.97	2.71	3.548 (3)	145
C11—H*A*···Cl1^ii^	0.97	2.71	3.548 (3)	145

Symmetry codes: (ii) *y*+1, −*x*+3/2, −*z*+1/2; (iv) −*y*+3/2, *x*−1, −*z*+1/2.
